# *Toxoplasma gondii* parasites induce a localized myeloid cell immune response surrounding parasites in the brain during acute infection

**DOI:** 10.1128/mbio.00810-25

**Published:** 2025-06-10

**Authors:** Stephanie B. Orchanian, Sebastian Zolog, Julia Tomasello, Rijul Malik, Ji-Hun Shin, Anita A. Koshy, Melissa B. Lodoen

**Affiliations:** 1 Department of Molecular Biology and Biochemistry, University of California 8788, Irvine, California, USA; 2 Institute for Immunology, University of California 8788, Irvine, California, USA; 3 Neuroscience Graduate Interdisciplinary Program, University of Arizona 8041, Tucson, Arizona, USA; 4 Department of Immunobiology, University of Arizona 8041, Tucson, Arizona, USA; 5 Bio5 Institute, University of Arizona 8041, Tucson, Arizona, USA; 6 Department of Neurology, University of Arizona 8041, Tucson, Arizona, USA; UT Southwestern Medical Center, Dallas, Texas, USA

**Keywords:** *Toxoplasma gondii*, monocyte, brain, neuroinflammation, myeloid cells, immune response

## Abstract

*Toxoplasma gondii* is a foodborne intracellular parasite that chronically infects the brain. Although *T. gondii* infection elicits a protective immune response, the nature of the monocyte response in the immediate vicinity of parasites during initial brain infection is not well understood. By infecting mice with *T. gondii* and comparing areas of the brain containing or not containing parasites, we found an increase in CCR2^+^ monocytes, IBA1^+^ myeloid cells, GFAP^+^ astrocytes, and CD68^+^ signal near parasites in the brain, indicating immune cell infiltration and phagolysosomal activation in response to the infection. CCR2^+^CD68^+^ monocytes were specifically increased near tachyzoites with minimal localization of these cells near cysts in the brain. This monocyte response was also detected near parasite-interacted cells, identified using *T. gondii* parasites that inject Cre recombinase into “interacted” cells of Ai6 CCR2^RFP/+^ mice, enabling us to track these events *in vivo*. The chemokine CCL2 and its transcription factor NF-κB were also upregulated surrounding parasites in the brain; however, the *T. gondii* effector protein GRA15, which sustains NF-κB activation in infected cells, was not required for CCL2 production, NF-κB activation, or myeloid cell recruitment to parasites in the brain. In contrast, active *T. gondii* replication played a more significant role, as CCR2^+^ monocytes were recruited to replication-competent but not replication-deficient *T. gondii* delivered via intracranial injection into mice. These findings provide novel insights into the drivers of immune cell mobilization and activation in the brain during initial *T. gondii* central nervous system infection and highlight the importance of parasite replication in this process.

**IMPORTANCE:**

*Toxoplasma gondii* is a brain-infecting parasite, and the mobilization of peripheral immune cells to the brain is critical for controlling *T. gondii* infection. However, the initial events driving these cells to sites of *T. gondii* infection in the brain are poorly understood. We show that peripheral myeloid immune cell recruitment and activation are specific to areas of the brain containing actively replicating parasites, which are capable of lysing host cells. This local immune response is characterized by focal chemokine production, myeloid cell recruitment, and the activation of phagolysosomal pathways. These highly localized host responses were independent of the parasite effector protein that induces NF-κB activation within infected cells, GRA15. However, the localized monocyte recruitment was dependent on live parasites and active parasite replication. This research highlights the importance of host cell sensing of parasite replication in the brain for immune control of *T. gondii* infection.

## INTRODUCTION

The brain is a unique organ due to its tightly regulated blood-brain barrier (BBB) that restricts the passage of cells, molecules, and toxins from the circulation into the brain (1). As a result, few pathogens can breach the BBB to enter the brain (2). Those that do pose a unique challenge for the immune response, since inflammatory responses in the brain may adversely affect neuronal function. Pathogens that cause a chronic infection and persist in the brain must be able to establish a niche and evade immune clearance, which may be facilitated by transitioning into a dormant, latent, or non-replicative state. A successful immune response against such microbes, therefore, selectively targets infected areas of the brain while minimizing collateral neuronal damage.

*Toxoplasma gondii* is one of the rare pathogens that can enter and infect cells in the brain (3). Transmitted via ingestion of contaminated food or water, or via congenital infection, *T. gondii* is an obligate intracellular parasite that infects approximately 30% of the world’s human population and can infect any nucleated cell within virtually any warm-blooded animal (4, 5). During acute infection, *T. gondii* has a lytic cycle, replicating within host cells in the tachyzoite form (6). After *T. gondii* enters the body, it traverses the intestinal epithelium to enter the circulation and infiltrates the brain during the acute phase of the infection (7). *T. gondii* may enter the brain through a variety of different mechanisms, including the infection and lysis of the vascular endothelium at the BBB (8) or choroid plexus (9), or by infecting infiltrating immune cells that traffic to or across the BBB (10, 11). Once in the brain, the parasites undergo stage conversion to the slow-growing bradyzoite form and establish a chronic infection within cysts in infected neurons. This differentiation into cyst-forming bradyzoites facilitates the parasites’ ability to resist immune clearance in the brain (12). However, in the event of recrudescent infection, the parasites differentiate back into tachyzoites with a lytic life cycle, which, along with the ensuing host response to the parasite, can lead to the formation of large lesions in the brain and potentially death (13).

In immunocompetent hosts, the immune response is initiated by recognition of *T. gondii* by Toll-like receptors (TLRs) and a subsequent protective adaptive immune response. TLR2, 4, 7, 9, 11, and 12 have all been implicated in innate recognition of *T. gondii*, with TLR11 and TLR12 specifically expressed in rodents (14). TLR recognition of *T. gondii* leads to interleukin-12 (IL-12) secretion by dendritic cells, which drives interferon-gamma (IFN-γ) production by natural killer cells and T helper 1 cells (15–17). IFN-γ then induces a cascade of antimicrobial pathways, including expression of inducible nitric oxide synthase, indoleamine 2,3-dioxygenase, immunity-related guanosine triphosphatase, and guanylate-binding proteins in infected host cells (18, 19), which lead to starvation of *T. gondii* or disruption of the parasitophorous vacuole (19–22). However, *T. gondii* can also differentiate into cyst-forming bradyzoites (23), which persist for the remainder of the host’s life span (24, 25). When bradyzoites reactivate and convert back into the highly replicative, lytic tachyzoite form, the infection can become fatal, as is the case in AIDS-related toxoplasmic encephalitis (26).

With the high global prevalence of *T. gondii* as an opportunistic parasite and its potential to cause fatal encephalitis, there have been considerable efforts to understand the host immune response in the brain and the drivers of protective immunity or pathology. As *T. gondii* parasites enter the brain during acute infection, the choroid plexus becomes an early site of neuroinflammatory responses, with elevated proinflammatory cytokine and chemokine production (9). Peripheral immune cells, including lymphocytes and monocytes, also mobilize to the brain in significant numbers and are recruited in a brain region-specific manner (27–29). Type I innate lymphoid cells mobilize to the brain parenchyma, choroid plexus, and meninges during early acute infection, produce IFN-γ and tumor necrosis factor α (TNF-α), and are critical for initiating host defense in the brain (29). Monocytes also play a key role in the control of *T. gondii* infection in the brain (28) and the periphery (30, 31). We recently found that by 15 days after intraperitoneal (i.p.) infection with *T. gondii*, MAC2^+^ myeloid cells are specifically increased in areas of the brain containing tachyzoites (32). During chronic infection, parasites stage-convert into the cyst-forming bradyzoites, and Ly6G^+^ neutrophils and chitinase-expressing macrophages can be found adjacent to cysts (33, 34), whereas T cells survey tachyzoite-infected cells (35, 36). T cells also appear to cluster tightly around parasite-interacted neurons in the brain (37), and major histocompatibility complex (MHC) class I presentation of parasite antigens by neurons and microglia regulates the differentiation of *T. gondii*-specific brain-resident CD8^+^ T cells (38). Perivascular cuffing by mononuclear cells has also been observed in chronic infection (39). During parasite reactivation, IBA1^+^ myeloid cells (which include microglia, macrophages, and mature monocytes) and GFAP^+^ astrocytes can be found near reactivation events (40). Therefore, there appear to be local immune responses specifically near sites of *T. gondii* infection. Outside of the central nervous system (CNS), many factors have been identified that drive immune cell recruitment to *T. gondii*, including parasite pathogen-associated molecular patterns (PAMPs), such as profilin, and host chemotactic factors (28, 31, 41). However, there is a limited understanding of how the initial monocyte response to replicating *T. gondii* tachyzoites in the brain is elicited.

This study focuses on defining the neuroimmune response to sites of *T. gondii* parasites in the brain during acute infection and identifying the factors involved in this response. We observed an increase in activated myeloid immune cells (IBA1^+^, CCR2^+^, and CD68^+^ signal) specifically in the vicinity of *T. gondii* in the brain. These myeloid cells, as well as CCL2- and NF-κB-producing cells, were in close proximity to the parasites; however, the localized myeloid cell response surrounding parasites was independent of the NF-κB-inducing *T. gondii* GRA15 protein. Instead, active parasite replication proved to be an important factor in driving this response, as activated inflammatory monocytes were selectively enriched at sites of tachyzoites compared to cysts. Intracranial (i.c.) injection of mice with live, replication-competent *T. gondii*, but not heat-killed or UV-treated parasites nor phosphate-buffered saline (PBS), resulted in significant immune cell recruitment to infected brains, indicating a robust response only to replicative parasites. This research characterizes the host response to sites of *T. gondii* infection in the brain and highlights the importance of active parasite replication in driving highly localized myeloid immune cell recruitment during acute infection.

## RESULTS

### Myeloid cell activation in the brain is localized to sites of *T. gondii* parasites

**Fig 1:**
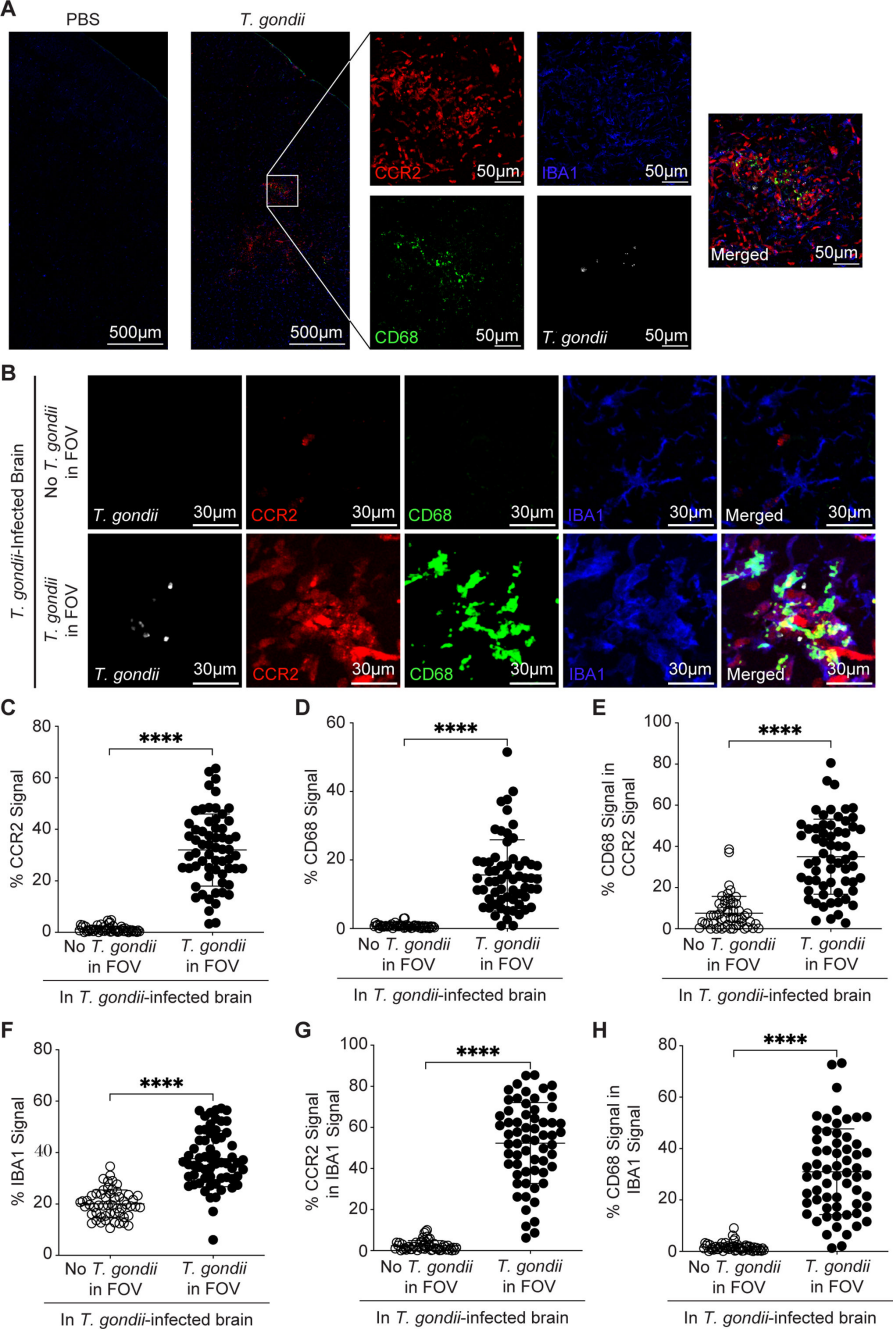
*T. gondii* infection induces CCR2^+^ monocyte recruitment and activation surrounding parasites in the brain. CCR2^RFP/+^ mice were i.p. injected with PBS or type II *T. gondii*, and brains were harvested and examined at 15 DPI. (**A**) Representative tile scan of *T. gondii* (white)*,* CD68^+^ cells (green), CCR2^+^ cells (red), and IBA1^+^ cells (blue) in brains of infected or PBS-injected control mice. Magnified inset shows a FOV with *T. gondii* parasites. (**B**) Representative confocal microscopy of *T. gondii* (white)*,* CD68^+^ cells (green), CCR2^+^ cells (red), and IBA1^+^ cells (blue) in brains of infected mice in FOVs containing *T. gondii* (bottom row) or >1,500 µm away from *T. gondii* (top row). Quantification of the percent area per FOV of CCR2^+^ signal (**C**), CD68^+^ signal (**D**), CD68^+^ signal within CCR2^+^ area (**E**), IBA1^+^ signal (**F**), CCR2^+^ signal within IBA1^+^ signal (**G**), or CD68^+^ signal within IBA1^+^ signal (**H**) in FOVs containing parasites or >1,500 µm away from *T. gondii* in infected brains. In panels **C through H***, n* = 55–62 FOVs within the cerebrum, midbrain, and interbrain from 13 mice per group from three independent experiments. Statistical significance was determined by Student’s *t*-test in panel F or Mann-Whitney *U* test in panels C, D, E, G, and H. ****P < 0.0001.

To investigate the immune response surrounding *T. gondii* tachyzoites in the brain during acute infection, CCR2^RFP/+^ mice were i.p. injected with PBS as a control or with green fluorescent protein (GFP)-expressing type II *T. gondii*. In the CCR2^RFP/+^ mice, one allele of *ccr2* is replaced with red fluorescent protein (RFP) under the control of the *ccr2* promoter, such that peripheral and tissue-infiltrating CCR2^+^ monocytes express RFP (42). During *T. gondii* acute infection of the brain, over 90% of RFP^+^ cells in these mice are comprised of monocytes (32). At 15 days post-infection (DPI), the mice were perfused to remove adherent blood cells, and brain sections were imaged in 100 µm × 100 µm fields of view (FOVs) containing parasites or in FOVs without parasites (and at least 1,500 µm away from parasites) within the cerebrum, midbrain, and diencephalon. Unlike in PBS-injected mice, in brains from *T. gondii-*infected mice, we observed the infiltration of CCR2-RFP cells and expression of CD68, the phagolyosomal protein macrosialin (Fig. 1A), which is upregulated in activated myeloid cells (43, 44). Notably, CCR2-RFP cells and CD68-expressing cells were significantly increased in FOVs containing parasites and rarely detectable in FOVs without parasites in the *T. gondii-*infected brains (Fig. 1B through D). Approximately 35% of the CD68 signal was found within CCR2^+^ cells (Fig. 1E). By staining brain sections from infected mice for the myeloid cell marker IBA1, we also observed a significant increase in IBA1^+^ cells in FOVs with *T. gondii* compared to those without *T. gondii* (Fig. 1A, B, and F), with ~50% of IBA1^+^ signal overlapping with CCR2^+^ signal (Fig. 1G). The increase in CCR2^+^ cells in the *T. gondii-*infected brain also occurs during chronic *T. gondii* infection (28). Additionally, in FOVs containing parasites, both CCR2^+^ cells and IBA1^+^ cells were more likely to express CD68 (Fig. 1E and H). These data indicate focal monocyte infiltration and activation, based on CD68 expression, that is specific to areas of the brain in the immediate vicinity of *T. gondii* parasites.

**Fig 2:**
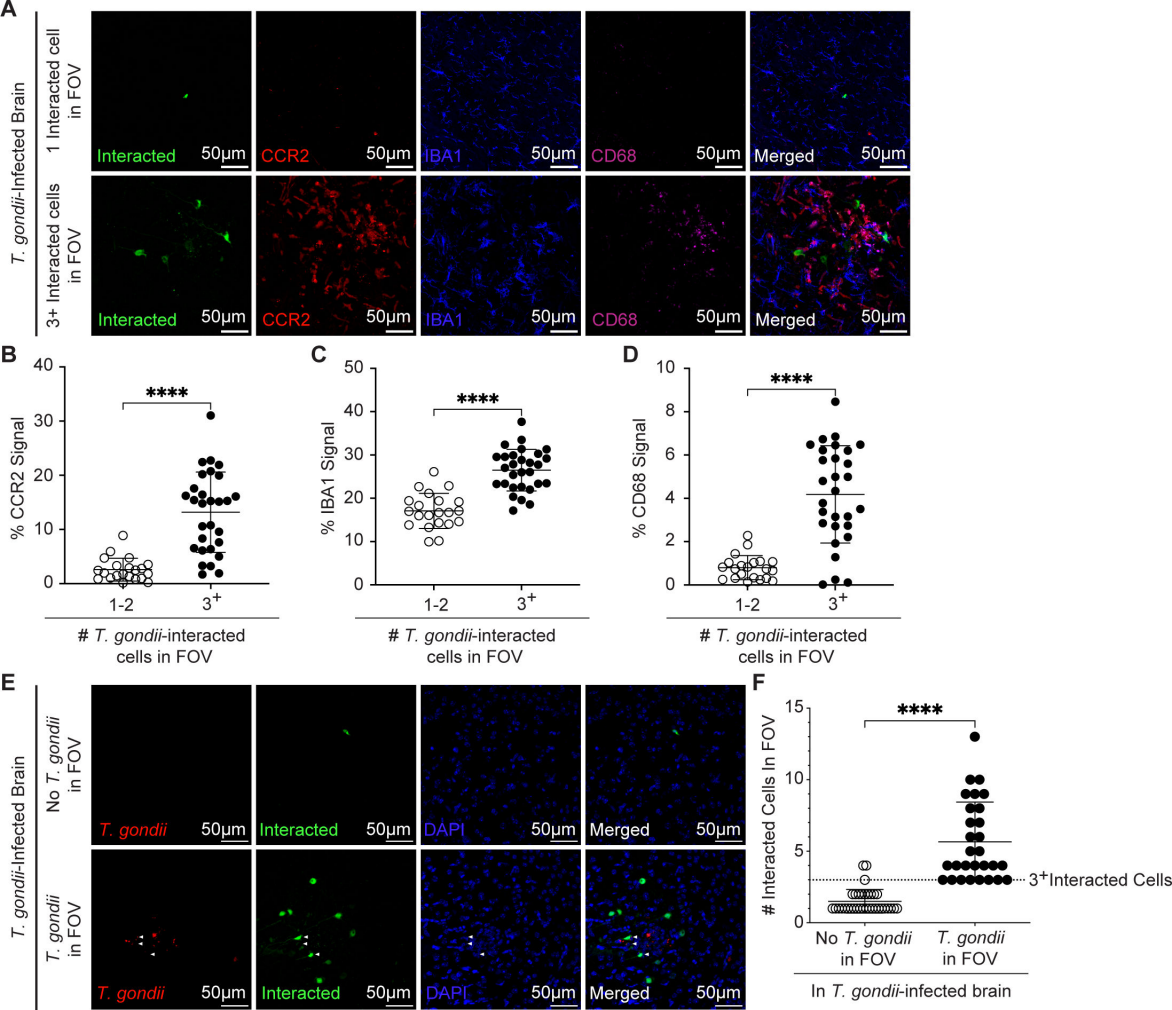
*T. gondii*-interacted cells are detected near tachyzoites and activated myeloid cells. Ai6 heterozygous CCR2^RFP/+^ mice were infected with type II toxofilin-Cre *T. gondii*, and brains were harvested and examined at 15 DPI. (**A**) Representative confocal microscopy of *T. gondii-*interacted cells (green), CCR2^+^ cells (red), IBA1^+^ cells (blue), and CD68^+^ cells (magenta) in brains of infected mice in FOVs containing one *T. gondii*-interacted cell (top row) or three or more (3+) *T. gondii*-interacted cells (bottom row) in infected brains. Quantification of the percent area per FOV of CCR2^+^ signal (**B**), IBA1^+^ signal (**C**), or CD68^+^ signal (**D**) in FOVs containing parasites or >1,500 µm away from *T. gondii* in infected brains. (**E**) Representative confocal microscopy of *T. gondii* (red), *T. gondii-*interacted cells (green), and DAPI (blue) in brains of infected mice in FOVs containing parasites (bottom row) or >1,500 µm away from *T. gondii* (top row) in infected brains. (**F**) Quantification of total interacted cell number in FOVs containing parasites or >1,500 µm away from *T. gondii* in infected brains. In panels **B through D** *n* = 21–29 FOVs within the cerebrum, midbrain, and interbrain from 9 to 10 mice per group from two to three independent experiments. In panel **F** *n* = 29–34 FOVs within the cerebrum, midbrain, and interbrain from 9 mice per group from three independent experiments. Statistical significance was determined by Student’s *t*-test in panel C or Mann-Whitney *U* test in panels B, D, and F. ****P < 0.0001.

Interestingly, *T. gondii* can interact with and inject parasite effector proteins into host cells that it does not invade (45, 46). This process can alter the transcriptional landscape of injected cells, enabling the parasite to manipulate host cells that are not persistently infected (47). Within the brain, these injected cells highly outnumber infected cells and are mainly comprised of neurons (48). Additionally, transcripts associated with immune and inflammation-related pathways are upregulated near injected neurons from *T. gondii-*infected brains (37). To assess the degree to which CCR2-RFP cells are recruited to parasite-interacted cells, we bred CCR2^RFP/RFP^ mice to Ai6 mice. The Ai6 mice have a CAG promoter, *loxP*-flanked STOP cassette, and ZsGreen1 sequence inserted into the ROSA26 locus, such that Cre-mediated recombination deletes the STOP cassette and allows for high-level ZsGreen expression (49). The Ai6 het CCR2^RFP/+^ mice were injected i.p. with type II toxofilin-Cre parasites, in which Cre recombinase is fused to the secreted effector protein toxofilin and injected into interacted or infected host cells (50). As a result, parasite-interacted cells express ZsGreen, enabling their detection in the infected brain. These interacted cells include (i) infected host cells, (ii) host cells that were previously infected but killed the intracellular parasite, (iii) host cells that were injected with effector proteins but not infected (aborted invasion), and (iv) injected cells that underwent cell division and transferred ZsGreen expression to daughter cells but may no longer harbor parasites. Brain sections from *T. gondii-*infected mice were imaged in FOVs containing interacted cells (Fig. 2A). We found significantly greater CCR2-RFP signal, IBA1 signal, and myeloid cell activation (CD68^+^ cells) in FOVs containing three or more interacted cells compared to those with one to two interacted cells (Fig. 2B through D). To determine the relationship between the levels of interacted cells and the presence of *T. gondii*, Ai6 mice were i.p. injected with type II toxofilin-Cre parasites expressing mCherry and imaged by fluorescence microscopy at 15 DPI. We found an increase in interacted cells near parasites (Fig. 2E), supporting previous studies using this system (48). We detected interacted cells harboring the parasite mCherry signal (Fig. 2E, white arrowheads), but also several interacted cells without parasites (ZsGreen^+^mCherry^-^ cells). In addition, we observed mCherry^+^ parasites that were not within ZsGreen^+^ cells. These parasites may be extracellular, or they may have injected toxofilin-Cre into host cells that had not yet expressed the ZsGreen protein, as previously described (45, 48). Interestingly, in FOVs containing at least three interacted cells, greater than 90% of the FOVs (291 µm × 291 µm × 14 µm) also contained parasites (Fig. 2F). These data support the hypothesis that the increased CCR2-RFP cells and CD68-expressing cells were more significantly enriched in sites with a high level of parasite infection and interaction with host cells.

### NF-κB p65 and CCL2 are increased near parasites in the brain independent of GRA15

**Fig 3:**
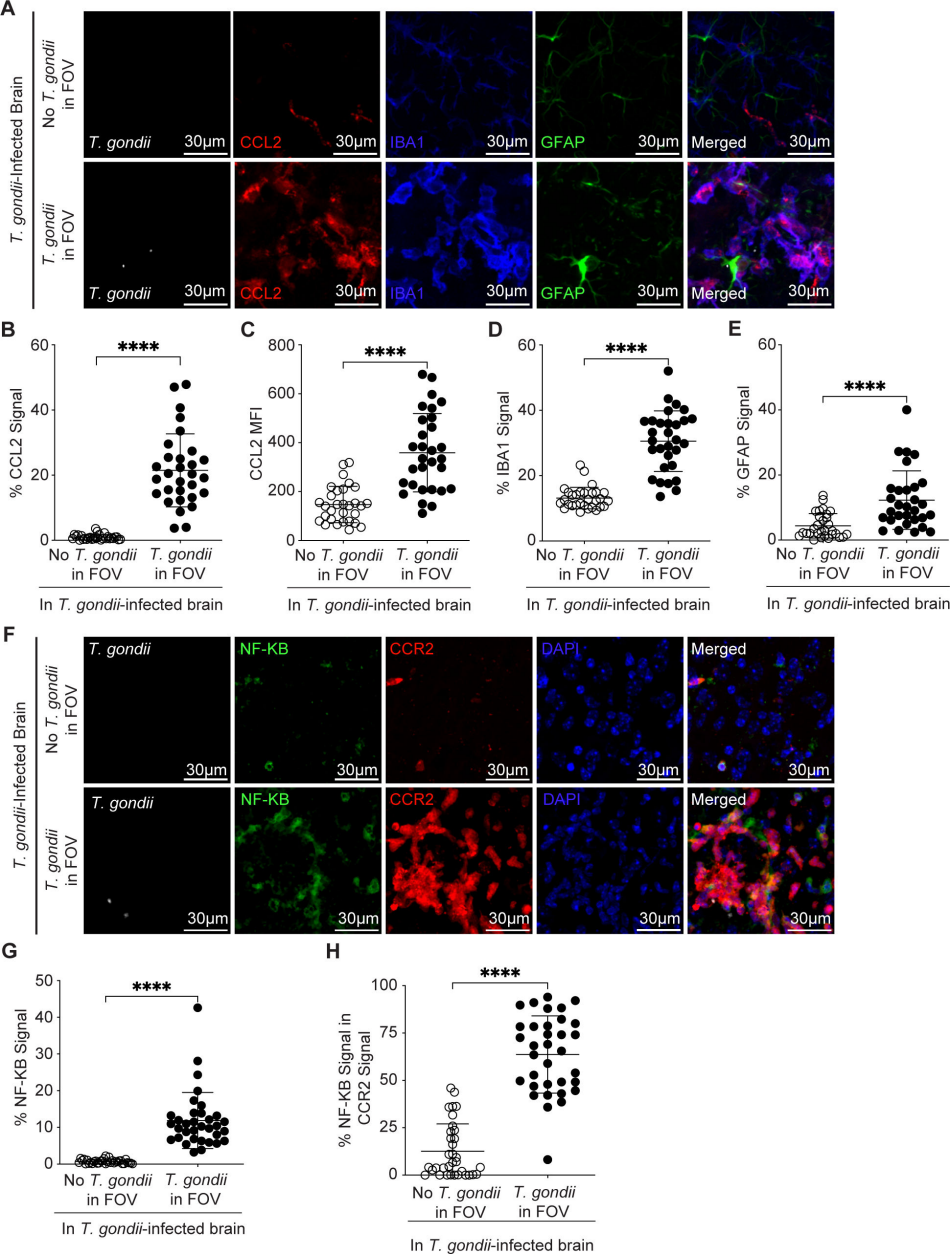
*T. gondii* infection induces NF-κB and CCL2 production near parasites. CCL2-RFP mice were infected with type II *T. gondii*, and brains were harvested and examined at 15 DPI. (**A**) Representative confocal microscopy of *T. gondii* (white)*,* GFAP^+^ cells (green), CCL2^+^ cells (red), and IBA1^+^ cells (blue) in brains of infected mice in FOVs containing *T. gondii* (bottom row) or >1,500 µm away from *T. gondii* (top row). Quantification of the percent area of CCL2^+^ signal per FOV (**B**), MFI of CCL2^+^ signal (**C**), percent area of IBA1^+^ signal per FOV (**D**), and percent area of GFAP^+^ signal per FOV (**E**) in FOVs containing parasites or >1,500 µm away from *T. gondii* in infected brains. (**F**) Representative confocal microscopy of *T. gondii* (red)*,* NF-κB^+^ cells (green), and DAPI (blue) in brains of infected mice in FOVs containing *T. gondii* (bottom row) or >1,500 µm away from *T. gondii* (top row). Quantification of the percent area of NF-κB^+^ signal per FOV (**G**) or the percent area of NF-κB^+^ signal within CCR2^+^ signal (**H**) in FOVs containing parasites or >1,500 µm away from *T. gondii* in infected brains. In panels **B through E***, n* = 30 FOVs within the cerebrum, midbrain, and interbrain from seven mice per group from three independent experiments. In panels **G and H***, n* = 34 FOVs within the cerebrum, midbrain, and interbrain from 11 mice per group from three independent experiments. Statistical significance was determined by Student’s *t*-test in panel C or Mann-Whitney *U* test in panels B, D, E, G, and H. ****P < 0.0001.

Since the CCL2 chemokine is a potent chemoattractant for CCR2^+^ cells, we next examined the extent of CCL2 production in FOVs containing *T. gondii* and those not containing *T. gondii* in the brains of infected mice. CCL2-RFP reporter mice (51) were infected with GFP-expressing parasites as above, and brain sections were imaged by confocal microscopy. We detected a significant increase in CCL2-RFP signal in FOVs containing parasites, whereas little to no CCL2 signal was observed in FOVs without parasites (Fig. 3A). There was increased CCL2 area (Fig. 3B) and mean fluorescence intensity (MFI) of CCL2^+^ area (Fig. 3C) in FOVs containing *T. gondii*. The increased CCL2 signal was associated with increases in the cells that produce this chemokine, IBA1^+^ myeloid cells and GFAP^+^ astrocytes, near *T. gondii* in the brain (Fig. 3D and E). Since CCL2 is produced downstream of NF-κB signaling during immune activation, we next utilized confocal microscopy to measure the NF-κB p65 signal in the brain after staining the sections with an antibody that recognizes total p65, including both active and inactive p65 (52). We found NF-κB p65 signal to be increased, specifically in FOVs containing parasites (Fig. 3F and G), and 63.7% of the NF-kB signal was found within CCR2 signal (Fig. 3H). These data align with prior reports demonstrating a strong signature of NF-kB-dependent gene expression in infiltrating monocytes during *T. gondii* infection (53). Since we previously showed that CCR2^+^ cells produce CCL2 during acute *T. gondii* infection (32), we next sought to address whether parasite-induced NF-κB drives the focal CCL2 production in the brain.

**Fig 4:**
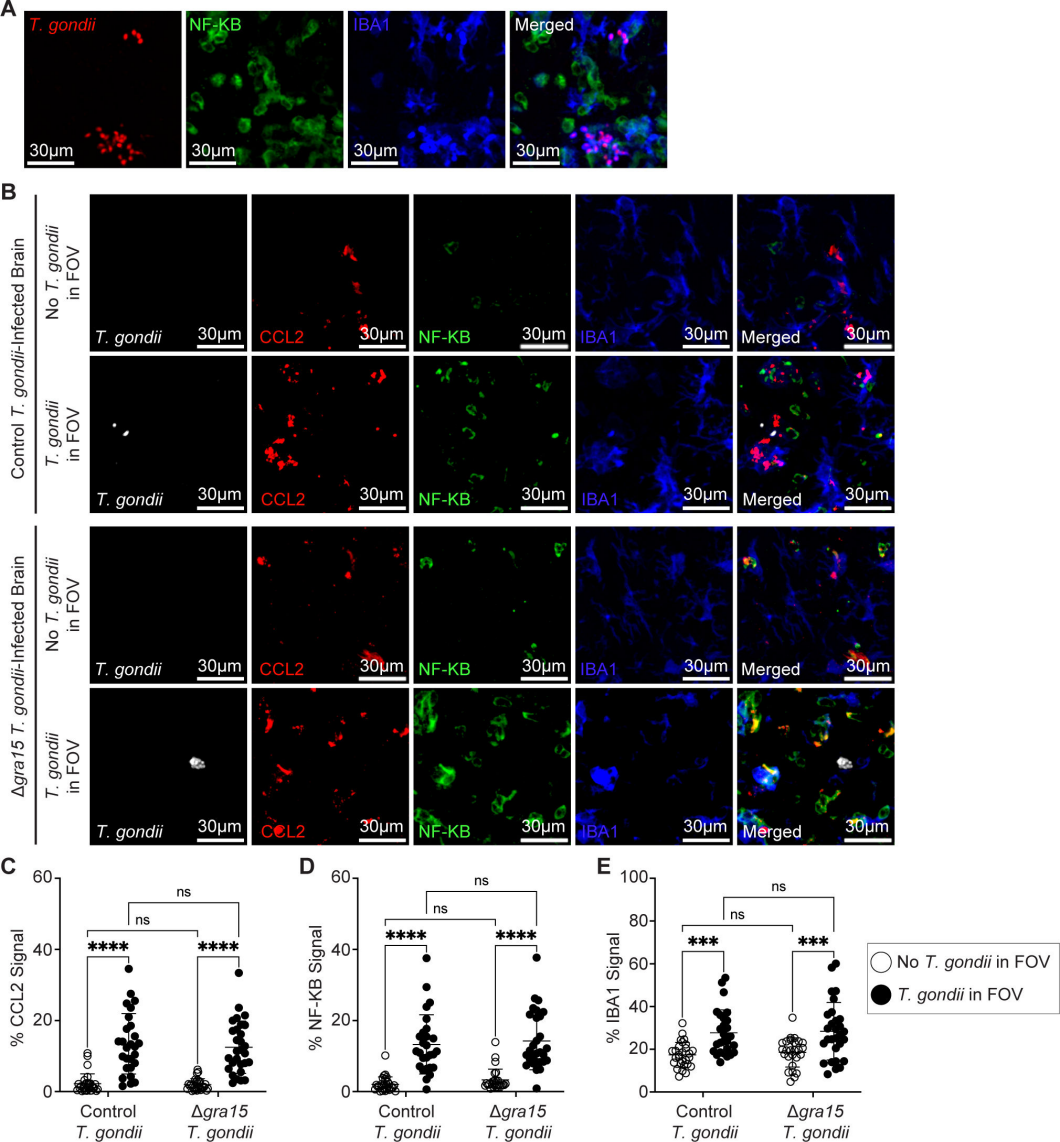
CCL2, NF-κB, and IBA1 signals are increased near *T. gondii* in the brain in a GRA15-independent manner. CCL2-RFP mice were infected with parental type II *T. gondii* or type II GRA15 knockout (∆*gra15*) *T. gondii*, and brains were harvested and examined at 15 DPI. (**A**) Representative confocal microscopy of *T. gondii* (red)*,* NF-κB^+^ cells (green), and IBA1^+^ cells (blue) in brains of mice infected with parental type II *T. gondii*. (**B**) Representative confocal microscopy of *T. gondii* (white)*,* CCL2^+^ cells (red), NF-κB^+^ cells (green), and IBA1^+^ cells (blue) in brains of mice infected with type II *T. gondii* (top two rows) or ∆*gra15 T. gondii* (bottom two rows) in FOVs containing *T. gondii* (bottom row) or >1,500 µm away from *T. gondii* (top row). Quantification of the percent area per FOV of CCL2^+^ signal (**C**), NF-κB^+^ signal (**D**), and IBA1^+^ signal (**E**) in FOVs containing parasites or >1,500 µm away from *T. gondii* in infected brains. In panels **C through E***, n* = 29–31 FOVs within the cerebrum, midbrain, and interbrain from six to eight mice per group from three to five independent experiments. Statistical significance was determined by a two-way analysis of variance. ***P < 0.001, ****P < 0.0001.

In addition to its activation by pattern-recognition receptors and cytokines, NF-κB signaling can be sustained by the *T. gondii* dense granule protein GRA15 (54). Since NF-κB p65 was observed in both uninfected and infected cells in the brain (Fig. 4A), we aimed to understand the extent to which GRA15 induced this localized NF-κB signal, CCL2 production, and myeloid cell recruitment to parasites in the brain. We examined these responses in brains from mice infected with the parental GFP-expressing *Prugniaud* strain parasites (55) or those infected with the GRA15 knockout (∆*gra15*) parasites (54). The ∆*gra15* parasites were confirmed to be deficient in *gra15* by PCR (Fig. S1), and they were also functionally deficient: they failed to induce IL-1β secretion from infected cells *in vitro*, unlike the control wild-type parasites (Fig. S2), as previously reported (56). In examining the brains of mice infected with control or ∆*gra15* parasites, we detected a significant increase in NF-κB, CCL2, and IBA1 in FOVs containing *T. gondii* compared to FOVs without parasites, but there were no differences between the parasite strains in any of these phenotypes (Fig. 4B through E). These data indicate that at this time point, NF-κB activation and CCL2 production in the vicinity of *T. gondii* in the brain were independent of GRA15. This outcome may be due to the relatively low number of infected cells producing NF-κB compared to the large number of infiltrating uninfected cells expressing this signaling protein.

### Parasite replication is necessary to induce CCR2^+^ cell recruitment to the brain

**Fig 5:**
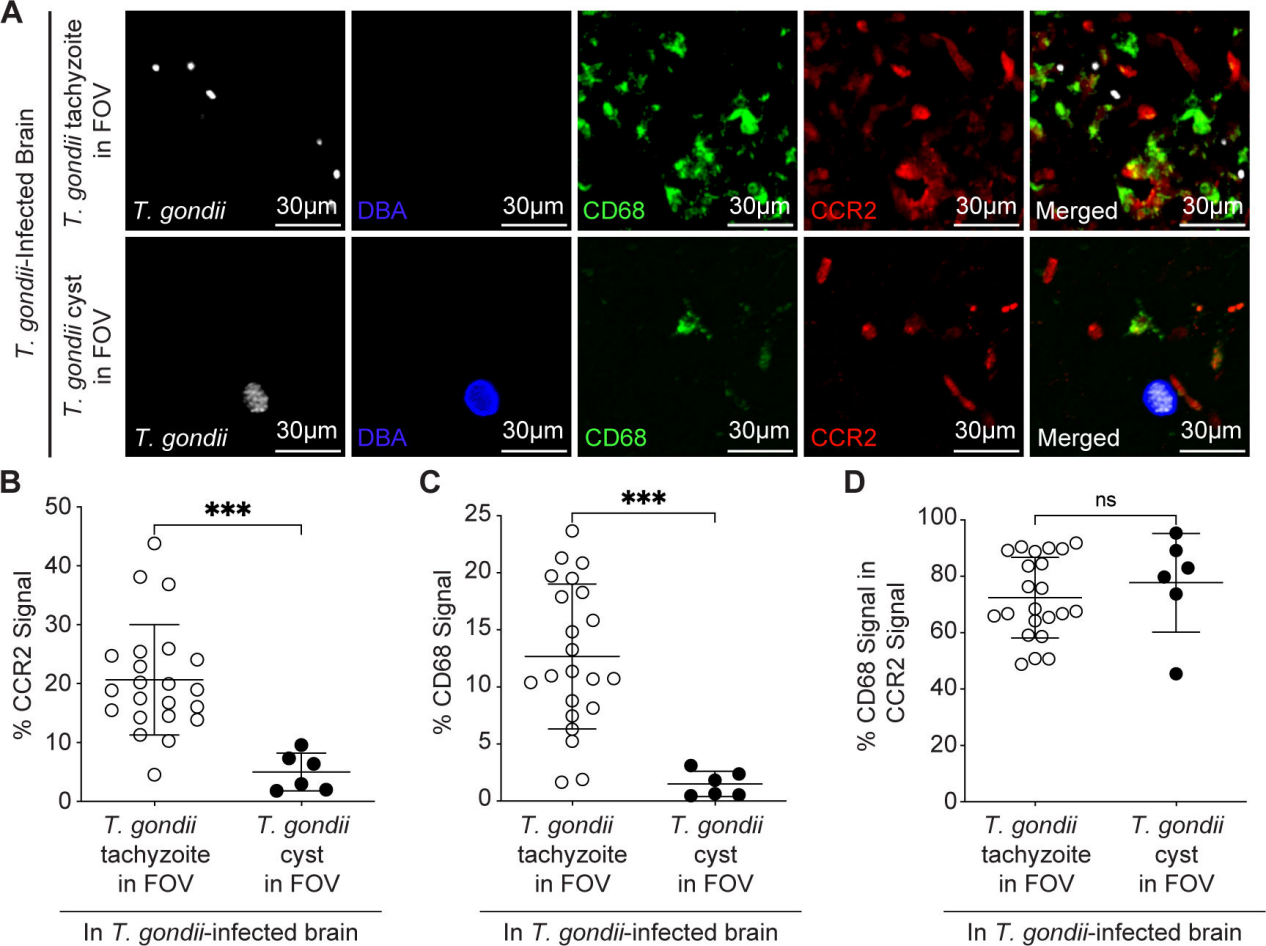
*T. gondii* tachyzoites, but not tissue cysts, induce activated CCR2^+^ monocytes surrounding parasites in the brain. CCR2^RFP/+^ mice were i.p. injected with type II *T. gondii*, and brains were harvested and examined at 28 DPI. (**A**) Representative confocal microscopy of *T. gondii* (white)*, Dolichos biflorus* agglutinin (DBA; blue), CD68^+^ cells (green), and CCR2^+^ cells (red) in brains of infected mice in FOVs containing *T. gondii* tachyzoites (top row) or a cyst (bottom row). Quantification of the percent area per FOV of CCR2^+^ signal (**C**), CD68^+^ signal (**D**), or CD68^+^ signal within CCR2^+^ area. In panels **B through D***, n* = 6–22 FOVs within the cerebrum, midbrain, and interbrain from 5 mice from three independent experiments. Statistical significance was determined by Student’s *t*-test. ***P < 0.001.

The lytic cycle of *T. gondii* results in the release of PAMPs and damage-associated molecular patterns (DAMPs) into the tissue surrounding infected cells during parasite egress. Indeed, we detected an increase in CCR2^+^ cells and in cell death (via cleaved caspase-3) in FOVs containing parasites in the brains of *T. gondii-*infected mice (Fig. S3). We therefore hypothesized that the localized monocyte response to sites of parasitic infection in the brain may be due in part to parasite replication and subsequent cell death events. To investigate this possibility, we examined the myeloid cell responses to replicating tachyzoites compared to the bradyzoite-containing cysts (as identified by *Dolichos biflorus* agglutinin [DBA]) at 28 DPI in CCR2^RFP/+^ mice. Notably, the levels of CCR2-RFP cells and CD68-expressing cells were significantly increased in FOVs containing tachyzoites compared to FOVs containing cysts in the *T. gondii-*infected brains (Fig. 5A through C). However, CCR2^+^ cells were equally likely to express CD68, whether in the presence of tachyzoites or cysts (Fig. 5D), indicating increased phagolysosomes in CCR2^+^ cells adjacent to both stages of the parasite.

**Fig 6:**
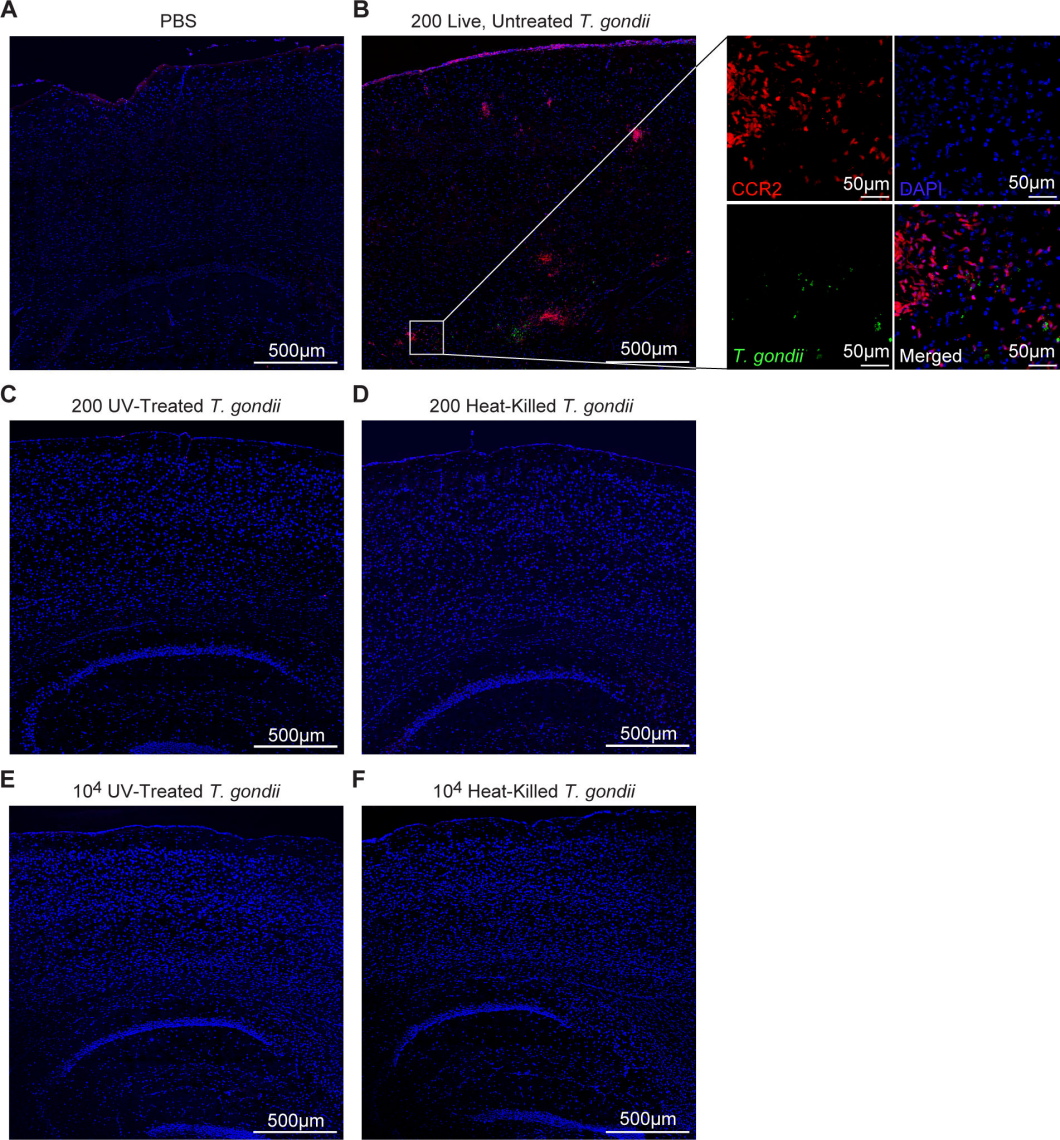
Intracranial infection with live, replicating *T. gondii* induces CCR2^+^ monocyte recruitment to the site of injection. CCR2^RFP/+^ mice were intracranially injected in the V1 region of the cortex with *T. gondii* tachyzoites or PBS as a control, and brains were harvested and examined at 7 DPI. Representative confocal microscopy of *T. gondii* (green)*,* CCR2^+^ cells (red), DAPI (blue) at the injection site in mice treated with PBS (**A**) or 200 live, untreated (replication-competent) *T. gondii* parasites (**B**), 200 UV-treated *T. gondii* parasites (**C**), 200 heat-killed *T. gondii* parasites (**D**), 10^4^ UV-treated *T. gondii* parasites (**E**), or 10^4^ heat-killed *T. gondii* parasites (**F**). Magnified inset shows a FOV from a brain injected with live, untreated *T. gondii* parasites.

To further examine the importance of parasite replication in CCR2^+^ cell recruitment to *T. gondii*, we next examined whether direct i.c. injection of *T. gondii* into mice induced CCR2^+^ monocyte recruitment to the brain. CCR2^RFP/+^ mice were i.c. injected with GFP-expressing type II *T. gondii* or PBS as a control. Injections were conducted into the cortex, where parasites are frequently detected during infection (57), in the stereotaxically accessible V1 region of the visual cortex. After 7 days, the mice were perfused to remove non-adherent blood cells, and the injected hemisphere was sectioned for imaging by confocal microscopy, or single cells were isolated for flow cytometric analyses. At 7 days after i.c. injection, PBS-injected control mice had few to no CCR2-RFP cells detectable in the brain near the injection site (Fig. 6A). In contrast, we observed a robust infiltration of CCR2-RFP cells in the *T. gondii-*infected mice at 7 DPI, and these cells were found in clusters throughout the cortex near GFP-expressing *T. gondii* (Fig. 6B). We also detected CCR2-RFP cells and GFP^+^ parasites in the leptomeninges of these mice (Fig. 6B). To determine whether the CCR2-RFP cells were responding to host cell sensing of parasite antigens by brain-resident cells or to the effects of parasite replication and host cell lysis, we compared the response in the brain after i.c. injection of 200 live untreated *T. gondii*, heat-killed *T. gondii*, or UV-treated *T. gondii* parasites. Heat-killed *T. gondii* cannot infect host cells; however, heat-stable parasite antigens can still be recognized by host cells (41, 58). In contrast, UV-treated parasites can infect host cells, but due to the DNA damage resulting from UV treatment, these parasites have significantly reduced replication compared to untreated parasites and also decreased host cell lysis (59). We used these different infection conditions to examine the importance of parasite antigens (via heat-killed parasites) or parasite replication (via UV-treated parasites) in driving the CCR2^+^ monocyte response to *T. gondii* in the brain. By conducting confocal imaging of brain sections from these i.c.-infected mice, we found CCR2-RFP monocytes specifically recruited to the brains and meninges of the mice infected with live, replication-competent parasites (Fig. 6B), but not those infected with the same number of UV-treated (Fig. 6C) or heat-killed (Fig. 6D) parasites. We also examined the injected hemispheres at 3 DPI, to account for the possibility that the response to these parasites had resolved by 7 DPI. However, there were no increases in T cells (CD3^+^), neutrophils (Ly6G^+^), or monocytes (Ly6C^+^) with any of the parasite infection conditions compared to PBS-injected mice at 3 DPI (Fig. S4). Since replication-competent parasites expand in numbers during the 7 days after i.c. infection, whereas heat-killed and UV-treated parasites do not, we next sought to control for the effect of the differential parasite burden on monocyte infiltration of the brain during i.c. infection. To do this, we i.c. injected 10^4^ UV-treated or heat-killed *T. gondii* parasites into the brain. Interestingly, CCR2-RFP cells were virtually undetectable in the brains of mice injected with 10^4^ UV-treated (Fig. 6E) or heat-killed (Fig. 6F) parasites compared to i.c. infection with 200 live, replicating parasites (Fig. 6B).

**Fig 7:**
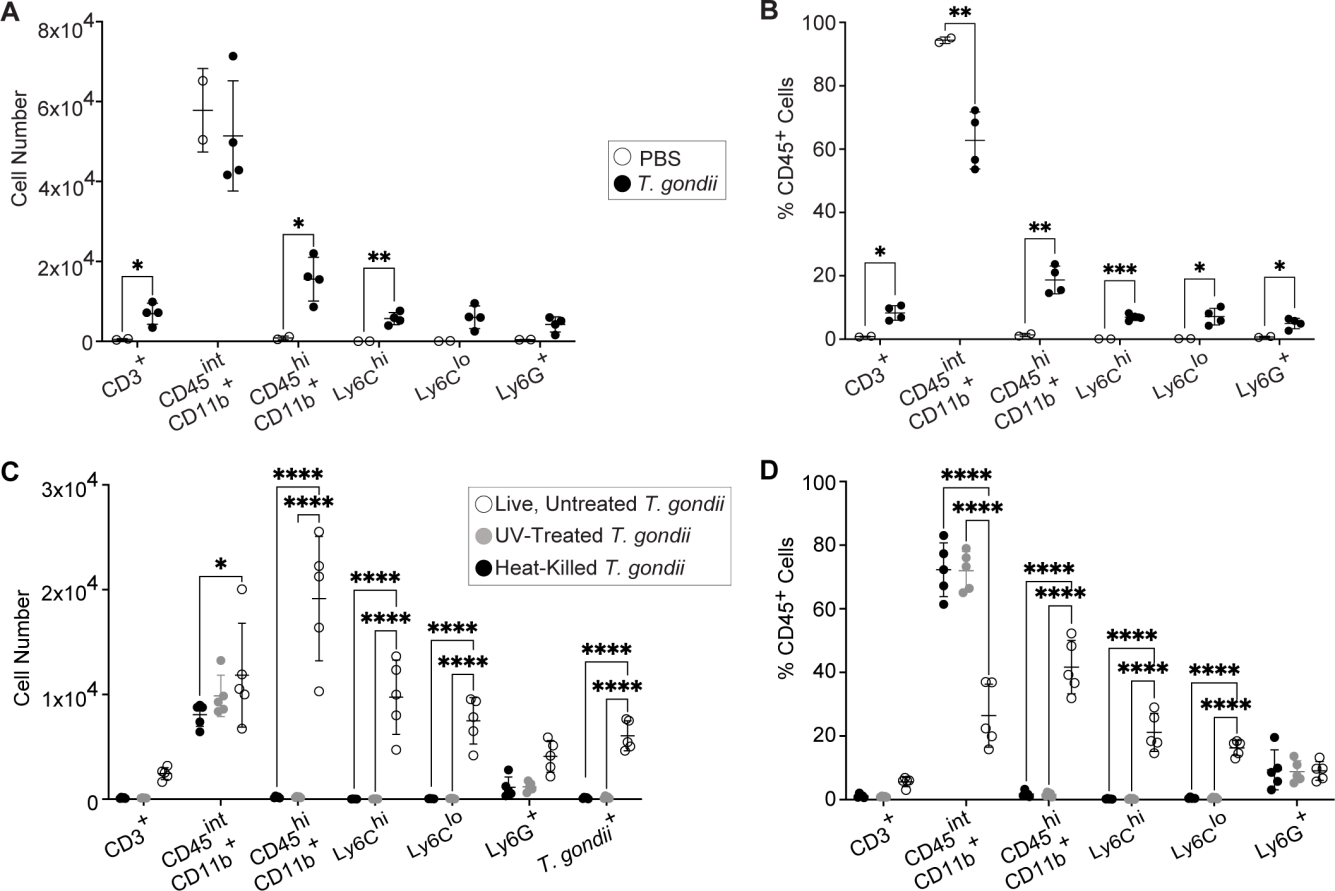
Monocytes and T cells are mobilized to the brain after intracranial infection with live, replicating *T. gondii*. (**A and B**) CCR2^RFP/+^ mice were intracranially injected in the V1 region of the cortex with 200 *T. gondii* tachyzoites or PBS as a control, and brain immune cell numbers (**A**) and frequencies of CD45^+^ cells (**B**) were quantified by flow cytometry 7 days later. (**C and D**) WT mice were intracranially injected with 200 heat-killed, UV-treated, or live, untreated *T. gondii* parasites, and brain immune cell numbers (**C**) and frequencies of CD45^+^ cells (**D**) were quantified by flow cytometry 7 days later. In panels **A and B**, *n* = 2–4 mice per group. Statistical significance was determined by Student’s *t*-test. In panels **C and D**, *n* = 5 mice per group. Statistical significance was determined by two-way analysis of variance. *P < 0.05, **P < 0.01, ***P < 0.001, ****P < 0.0001.

To more comprehensively examine the immune response to i.c. injected parasites, we conducted flow cytometric analysis of the infected hemispheres after i.c. injection of 200 live *T. gondii* parasites or an equivalent volume of PBS as a control. Consistent with the increase in CCR2-RFP monocytes observed by microscopy in the brains of *T. gondii-*infected mice (Fig. 6B), we observed a significant increase in the numbers and frequencies of T cells (CD3^+^), infiltrating myeloid cells (CD45^hi^CD11b^+^), and inflammatory monocytes (CD45^hi^CD11b^+^Ly6C^hi^) in mice injected with live parasites but not in PBS-injected mice (Fig. S5; Fig. 7A and B). This response was specific to replication-competent parasites, as heat-killed and UV-treated *T. gondii* did not elicit immune cell mobilization to the brain (Fig. 7C and D). As a control to ensure that our i.c. injection protocol did not cause a widespread systemic infection, we examined immune cell frequencies in the spleen. Unlike in the brain, most immune cell populations and subsets were unchanged in the spleens of mice i.c. injected with live untreated parasites, though we did observe a very small increase in myeloid cells and a decrease in CD4^+^ T cells (Fig. S6). These data indicate that i.c. injection of *T. gondii* predominantly results in immune cell infiltration of the brain without profoundly altering the composition of immune cells in the periphery (spleen) at 7 DPI. Collectively, these findings demonstrate that live, replicating parasites were necessary to mobilize peripheral immune cells, including T cells and CCR2^+^ monocytes, into the brain during infection.

## DISCUSSION

The neuroimmune landscape during *T. gondii* infection is defined by interactions among infiltrating immune cells, including myeloid cells and T cells, as well as brain-resident cells, including astrocytes and microglia, to control the infection. During the initial stages of *T. gondii* entry into the CNS, the parasites enter the choroid plexus and activate the nearby endothelial cells (9). The subsequent increase in the expression of inflammatory genes and decrease in tight junction proteins may aid in immune cell recruitment to the brain (9). However, how this response varies in the vicinity of parasites compared to more broadly in the brain and what factors drive this focal response are not well understood. Other studies have reported an increase in myeloid cells in the cortex and near interacted cells (28, 37). Indeed, we observed a robust increase in CD68^+^CCR2^+^ monocytes specifically near parasites and parasite-interacted cells in the brain. CD68 is present in phagolysosomal membranes (60), and its expression increases in cells during phagocytosis (61). The increase in CD68 signal near parasites is consistent with previous studies that have positively correlated parasite burden with cytokine production, another indicator of cellular activation (62). Since monocytes expressing CD68 were predominantly detected in parasite-infected areas of the brain, these data suggest that monocytes mobilized to sites of *T. gondii* infection upregulated phagocytic pathways, potentially to aid in parasite clearance. CD68, therefore, may serve as a useful indicator of antimicrobial activities in the brain. Notably, there is some evidence that certain strains of *T. gondii* can escape the phagosome and invade host cells via a process termed phagosome to vacuole invasion (63). Therefore, it is possible that the recruitment of these immune cells can also play a role in parasitic infection of host cells and dissemination through the brain (10).

NF-κB expression in CCR2^+^ monocytes was also observed, specifically near parasites, in the brain. This finding aligns with a prior study showing that infiltrating monocyte-derived macrophages express higher levels of genes associated with NF-κB signaling than microglia during the chronic stage of *T. gondii* infection (53). Since NF-κB signaling can drive CCL2 production, and monocytes express CCL2 (32), it is possible that NF-κB signal transduction contributes to maintaining local CCL2 production in parasite-infected areas of the brain. To decipher the role of parasite GRA15 in the localized NF-kB production near parasites, we conducted experiments using ∆*gra15* parasites. *In vitro*, GRA15 sustains NF-kB activation and plays a role in IL-12, IL-1β, and TNF-⍺ secretion in a variety of myeloid cells (54, 56, 64). *In vivo,* GRA15 contributes to IL-1 production in the brain during infection-induced seizure responses (65). However, we found similar levels of focal NF-κB p65, CCL2 expression, and IBA1^+^ cells near parasites in the brain after infection with control or ∆*gra15* parasites, indicating that these phenotypes occur in a GRA15-independent manner. This outcome may be due to the myriad activators of NF-κB in the infected brain, including PAMPs and DAMPs released from parasites and infected host cells. Moreover, GRA15 functions inside infected cells, and only a very small fraction of cells in the brain are infected. Therefore, GRA15 likely plays a less significant role in the overall NF-κB-dependent response when the parasite burden and number of infected cells are low, as is the case in this model of infection with *Prugniaud* parasites (27). Indeed, although ∆*gra15* parasites have elevated parasite burden in the periphery at 5 DPI after a high-dose infection in BALB/c mice (54), GRA15 does not influence parasite burden in the brain during subacute or chronic infection, when used at a lower dose in C57BL/6 mice (65, 66). Additional experiments focused on other effector proteins, specifically rhoptry proteins secreted into “interacted” host cells, which outnumber parasite-infected cells, may provide greater insights into parasite effector proteins that influence these local immune responses.

The comparison of monocyte responses near live, dead, and non-replicating tachyzoites as well as *T. gondii* cysts demonstrated the importance of the parasite replicative cycle and potentially host cell death in driving a monocyte response surrounding the parasites. These findings support the idea that the relative quiescence of cysts compared to tachyzoites facilitates the parasites’ ability to resist immune clearance in the brain (12). The TLR11/12 ligand profilin can induce immune responses in the periphery in mice (67); however, the recognition of *T. gondii* antigens in the absence of parasite replication and host cell lysis did not seem to be sufficient to mobilize immune cells to the brain. Indeed, the release of DAMPs from lysing cells may be a potent immune signal in the brain that amplifies the signals induced by parasite recognition. The alarmin IL-33 is upregulated in the brain during *T. gondii* infection, and the IL-33 receptor (ST2 or IL1RL1) plays a critical role in inducing neuroinflammation and controlling parasite burden (68). IL1RL1-expressing astrocytes contribute to host defense against *T. gondii* infection at 28 DPI, but interestingly, not at 12 DPI, a time point when IL1RL1 KO mice have increased levels of infiltrating myeloid cells (69). IL-33 therefore appears to enable immune cell homing to parasites once in the brain, but not in their mobilization to the brain itself. During chronic infection, IL-1⍺, which is released in a gasdermin-D-dependent manner from microglia, also contributes to immune cell recruitment to the brain (53). However, the degree to which IL-1⍺ functions in the highly localized immune response surrounding parasites during acute infection is unknown.

The intracranial model of infection has been used in other studies to understand *T. gondii*-host interactions (48), and our study builds on these prior findings to identify features of the parasite that contribute to a neuroinflammatory response surrounding the parasites. However, like all models, the i.c. injection model has its limitations: intracranial infection is not a physiological route of *T. gondii* infection, and the generation of a lesion in the meninges may increase the parasite’s ability to enter/exit the brain. It is also possible that the ensuing damage may drive a unique immune response not seen during an infection that originates in the periphery. The i.p. injection of *T. gondii* tachyzoites also has its own limitations, since the most common route of infection into humans is ingestion of cysts. In addition, i.p. infection with a low dose of the type II *Prugniaud* strain used in these studies (200 tachyzoites) results in a relatively low parasite burden in the brain compared to infection with ME49 strains. Finally, although mice are a natural host of *T. gondii* and a commonly used model for infection, the recognition of *T. gondii* in mice via TLR11/12 (58) does not occur in humans, as they do not express functional homologs of these TLRs. These limitations must be taken into consideration in regard to the translational potential of these findings to understanding human disease.

Collectively, these data describe the immune response localized to *T. gondii* in the brain and demonstrate that myeloid cells, CCL2 production, and NF-κB production are increased in focal areas near parasites in the brain. This study also defines a role for parasite replication and likely host cell lysis in recruiting immune cells to the parasites. This research contributes to our understanding of how the early immune response is orchestrated against *T. gondii* to control infection in the brain.

## MATERIALS AND METHODS

### Experimental mice

C57BL/6J (Jackson stock no. 000664), CCL2-RFP^fl/fl^ (Jackson stock no. 016849), Ai6 (Jackson stock no. 007906), and CCR2^RFP/RFP^ (Jackson stock no. 017586) mice were purchased from Jackson Laboratories. We bred C57BL/6J mice to CCR2^RFP/RFP^ mice to generate mice with CCR2^+^ cells that co-express CCR2 and RFP. We bred Ai6 mice to CCR2^RFP/RFP^ mice to generate mice with CCR2^+^ cells that co-express CCR2 and RFP and *T. gondii-*interacted cells that express ZsGreen.

Mice were infected with *T. gondii* and housed in ABSL2 facilities, separate from breeding animals. At necropsy, mice were anesthetized via intraperitoneal injection of 2.5% tribromoethanol (Avertin, Sigma Aldrich) and transcardially perfused with 50 mL of 1× PBS (Corning) to remove non-adherent blood cells.

For intracranial infections, 3D printed headplates were fixed onto a mouse skull using Vetbond (3M) and acrylic resin Ortho-Jet BCA (Lang Dental). Two days later, headplates were screwed into a stereotactic frame to stabilize the mouse, and then a 0.5 mm diameter burr hole was drilled into the skull of the mouse using a dental drill. To infect the mice, hand-pulled glass micropipettes with diameters of approximately 30 µm were used to inject 1 µL containing 1× PBS or 200 parasites in 1× PBS, at a fixed rate of 200 nL/min by using an automatic osmotic pump 1 mm anterior to the lambda, 3 mm lateral from the midline, and 0.5 mm down from the dural surface (within the V1 region of the isocortex). Mice were injected with carprofen prior to all surgical procedures (10 mg/kg of body weight subcutaneously).

### Parasite strains

Mice were infected intraperitoneally with 200 type II GFP-expressing *T. gondii* parasites, mCherry toxofilin-Cre-expressing *T. gondii* parasites, in which the rhoptry protein toxofilin is HA-tagged and fused to a nuclear localization signal and Cre recombinase (50), or GRA15 knockout *T. gondii* tachyzoites in 200 µL of 1× PBS. All type II parasites were from the *Prugniaud* strain. Tachyzoites were maintained by serial passaging in human foreskin fibroblasts, as previously described (55).

To generate heat-killed parasites, 200 or 10^4^ *T. gondii* parasites were isolated, and then placed in a heat block set to 100°C for 15 minutes, as previously described (56). To generate UV-treated parasites, 200 or 10^4^ type II *T. gondii* parasites were isolated, then UV-treated for 2 minutes in a UV Stratagene Stratalinker 1800, as previously described (59). We confirmed that this procedure resulted in viable parasites but little to no replication in human foreskin fibroblasts (HFFs) after 2 weeks of culture.

### Immunohistochemistry

After perfusion, brains were isolated and fixed in 4% paraformaldehyde for 4–12 hours at 4°C, cryoprotected in 30% sucrose at 4°C until the brain tissues sank, embedded in O.C.T. compound (Fisher), then stored at −80°C. Brains were cut into 14 µm-thick slices onto superfrost plus microscopy slides (Fisherbrand) using an HM525 NX Cryostat (Fisher Scientific). Sections were dried on the slides at 37°C then submerged in 1× PBS to remove O.C.T. Sections were stained at room temperature overnight in a humid chamber with primary antibodies (goat anti-mouse IBA1 Abcam cat#5076 1:50, rabbit anti-mouse IBA1 Wako cat#019-1974 1:500, rat anti-mouse GFAP Invitrogen cat#130300 1:300, rabbit anti-RFP Rockland cat#600-401-379 1:500, rat anti-RFP Proteintech Group Inc cat#5f8 1:500, rat anti-mouse CD68:Biotin Bio-Rad cat#MCA1957BT 1:400, rabbit anti-mouse NF-κB Abcam cat#ab16502 1:300, rabbit anti-mouse cleaved caspase-3 Cell Signaling cat#9661S 1:200), 3% donkey serum, and 0.3% Triton X-100 in 1× PBS. Sections were then submerged in 1× PBS to remove the primary antibody. Slides were then stained overnight at room temperature in a humid environment with secondary antibodies (donkey anti-goat Abcam cat#ab175665 1:500, donkey anti-rat Jackson Immunoresearch cat#712-606-153 1:500, donkey anti-rat Invitrogen cat#A-21209 1:500, donkey anti-rabbit Jackson Immunoresearch cat#711-585-152 1:500, streptavidin ThermoFisher cat#S32357 1:300, donkey anti-rabbit Abcam cat#ab150075 1:500), 3% donkey serum, and 0.3% Triton X-100 in 1× PBS. Sections were washed with 1× PBS followed by autoclaved water. Tissues were mounted using antifade mounting media (Vectashield) and covered with microscope cover glass (Fisherbrand). Slides were stored at 4°C. Images were collected with a TCS SP8 confocal microscope (Leica) and were limited to the cerebrum, midbrain, and interbrain (diencephalon). Images were analyzed using ImageJ software.

### Flow cytometry

After perfusion, mouse brains were isolated, then minced and digested using dispase II (Toche Applied Science) diluted in HEPES-buffered saline. The brains were triturated and passed through a 70 µm filter (Falcon). Myelin was removed using 1× PBS (Corning), 35% Percoll (GE Healthcare), and 75% Percoll gradients in which the cell layer between 35% Percoll and 75% Percoll was collected. To isolate single cells from spleens, the mouse spleens were minced and passed through a 40 µm filter (Falcon). Red blood cells were lysed using ammonium-chloride-potassium (ACK) lysing buffer (Gibco).

Prior to surface staining, the single cells were resuspended in 10% TrueStain FcX (Biolegend) in staining buffer (3% fetal bovine serum in 1× PBS) to inhibit non-specific binding. The cells were then stained with fluorescent dye-conjugated antibodies diluted in staining buffer for 30 minutes: from BioLegend: Ly6G:BV510 cat#127633, NK1.1:BV570 cat#108733, CD8⍺:BV650 cat#100742, CD11b:BV605 cat#101257, CD45:BV785 cat#103149, Ly6C:PerCp-Cy5.5 cat#128017, CD3ε:APC-Cy7 cat#100222, CD19:PE-Cy7 cat#115520, CD4:Pacific Blue cat#103417, and F4/80:APC cat#123116. Following staining, samples were resuspended in 1:1,000 propidium iodide (eBiosciences) in staining buffer and run on the Novocyte flow cytometer (Acea). Subsequently, the data were analyzed utilizing the FlowJo program (Tree Star).

### Statistical analyses

Normality in the data sets was determined using a Shapiro-Wilk test. Statistics were conducted using a Student’s *t*-test, Mann-Whitney *U* test, or two-way analysis of variance. Graphs were produced in Prism software version 10.1.1. The number of samples per group and statistical test used can be found in the figure legend. Statistics are represented as ns = not significant, *P < 0.05, **P < 0.01, ***P < 0.001, ****P < 0.0001.

## AFTER EPUB

*[This article was published on 10 June 2025 with errors in Results, the Fig. 5 legend, and Anita A. Koshy's name. The Results and figure legend were corrected on 12 June 2025, and the byline was corrected in the current version, posted on 26 June 2025.]*

## SUPPLEMENTAL MATERIAL

Supplemental material mbio.00810-25-s0001.pdf Supplemental figures and methods.

ASM does not own the copyrights to Supplemental Material that may be linked to, or accessed through, an article. The authors have granted ASM a non-exclusive, world-wide license to publish the Supplemental Material files. Please contact the corresponding author directly for reuse.
