# Supplementary material for: Toxoplasma gondii parasites induce a localized myeloid cell immune response surrounding parasites in the brain during acute infection
Source: mBio. 2025 Jun 10;16(7):e00810-25. doi: 10.1128/mbio.00810-25 (PMC12239593; doi:10.1128/mbio.00810-25)
Supplement: Supplemental material — Supplemental figures and methods. [file mbio.00810-25-s0001.pdf]

Orchanian *et al.*

Supplementary Information

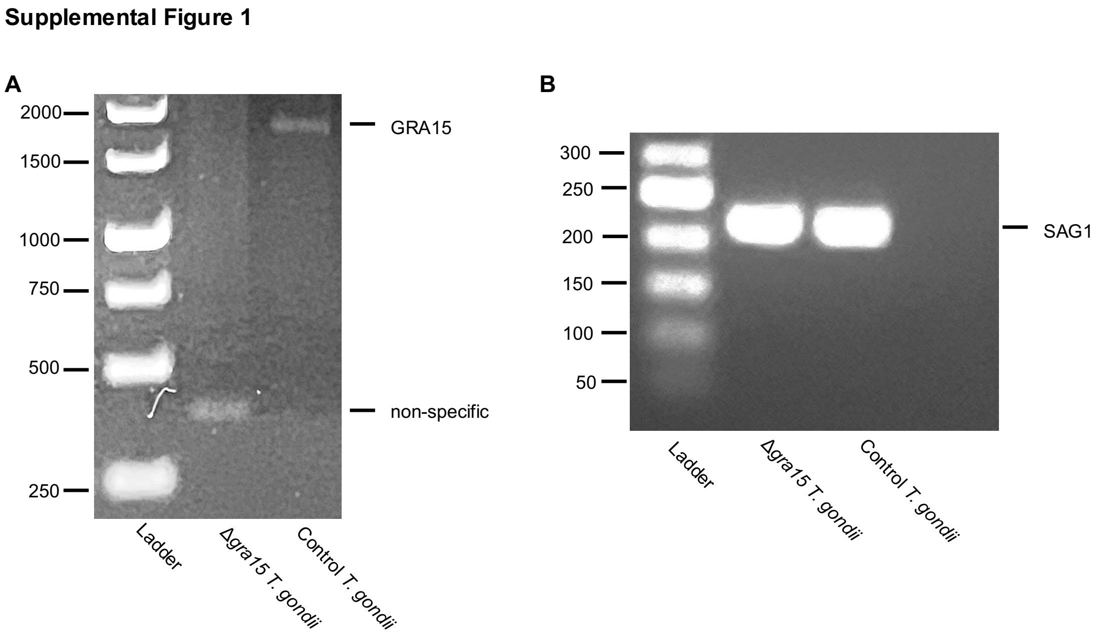

Figure S1. Confirmation of *gra15* knockout. Gel from PCR of genomic DNA from WT control parasites and Δ*gra15* parasites showing the presence of *gra15* in the control but not the knockout line (**A**), and of *sag1* (**B**) from both parasite lines.

Supplemental Figure 2

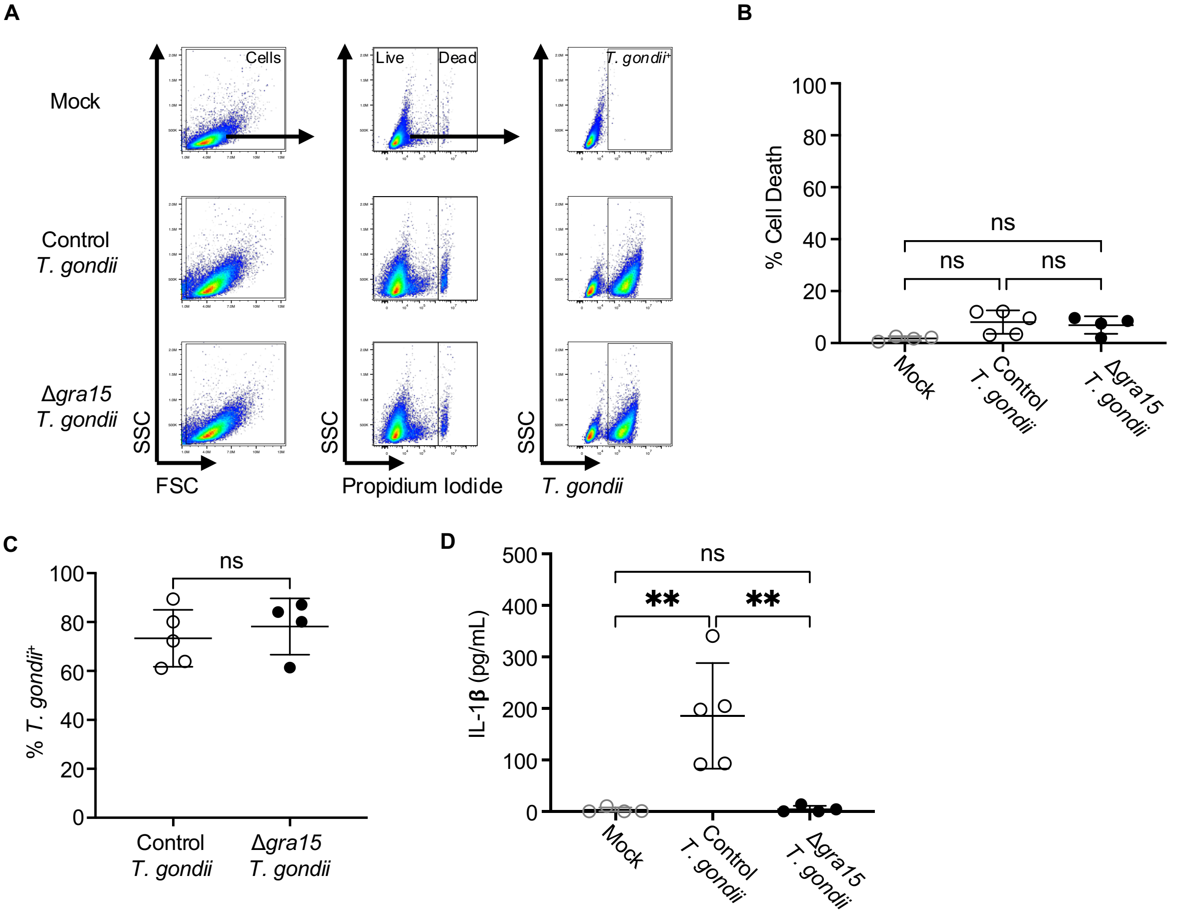

Figure S2. Δ*gra15* parasites produce less IL-1β than parental control *T. gondii* during infection. THP-1 cells were mock treated with media alone (mock) or infected with GFP-expressing control type II *T. gondii* or Δ*gra15 T. gondii* at a multiplicity of infection (MOI) of 2 for 18 hr. (**A**) Representative flow cytometry gating scheme of mock-treated or *T. gondii*-infected THP-1 cells after treatment with propidium iodide (PI) to identify dead cells. (**B**) Quantification of cell viability based on PI^+ cells. (**C**) Quantification of infection efficiency (percent of GFP^+ cells). (**D**) Quantification of IL-1β production by ELISA. Statistical significance was determined by Student's *t*-test in C or ANOVA in B and D. ***p*<0.01, ns: not significant.

Supplemental Figure 3

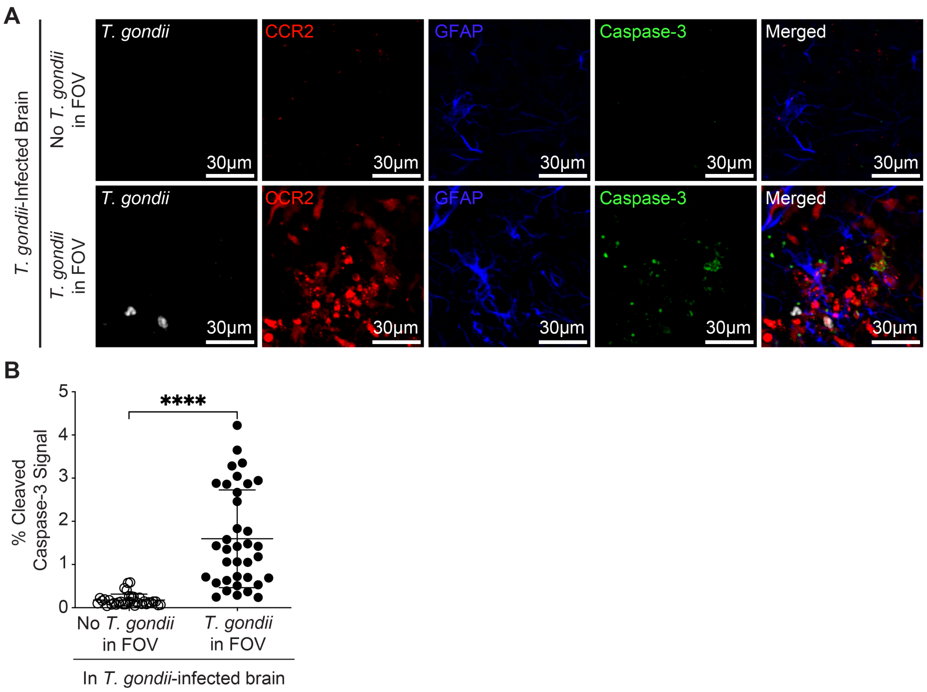

Figure S3. Cell death increases near *T. gondii* in infected brains. (**A**) Representative confocal microscopy of *T. gondii* (white), $CCR2^+$ cells (red), cleaved caspase-3^+ cells (green), and GFAP (blue) in brains of infected mice in FOVs containing *T. gondii* (bottom row) or ≥1,500 µm away from *T. gondii* (top row). (**B**) Quantification of the percent area of cleaved caspase-3 signal in FOVs containing parasites or ≥1,500 µm away from *T. gondii* in infected brains. n = 35-36 FOVs within the cerebrum, midbrain, and interbrain from 7 mice per group from 3 independent experiments. Statistical significance was determined by Mann-Whitney U test (B). ****=$p<0.0001$.

Supplemental Figure 4

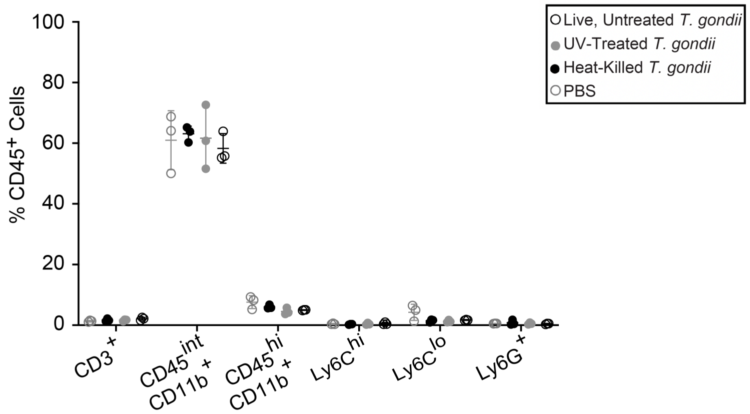

Figure S4. Composition of immune cells in the brains of mice three days after intracranial infection with *T. gondii*. WT mice were intracranially injected with PBS or with 200 type II live, untreated *T. gondii,* UV-treated *T. gondii,* or heat-killed *T. gondii*. Three days after infection, the injected hemisphere was harvested, and the frequencies of T cells ($CD3^+$), microglia ($CD45^{int}CD11b^+$), infiltrating myeloid cells ($CD45^{hi}CD11b^+$), inflammatory monocytes ($CD11b^+Ly6C^{hi}$), patrolling monocytes ($CD11b^+Ly6C^{lo}$), and neutrophils ($Ly6G^+$) cells out of the total $CD45^+$ cells were determined by flow cytometry. $n = 3$ mice per group. Error bars represent standard deviations.

Supplemental Figure 5

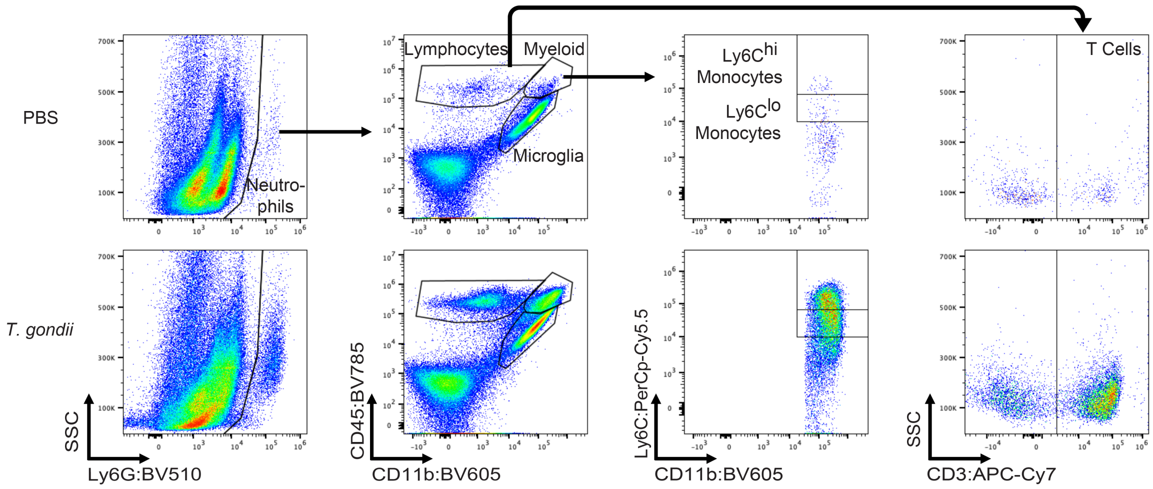

Figure S5. Gating strategies for immune cells in the brain. Representative flow cytometry gating scheme of brain immune cells from PBS-injected (top) or *T. gondii*-infected mice (bottom) at 7 DPI.

Supplemental Figure 6

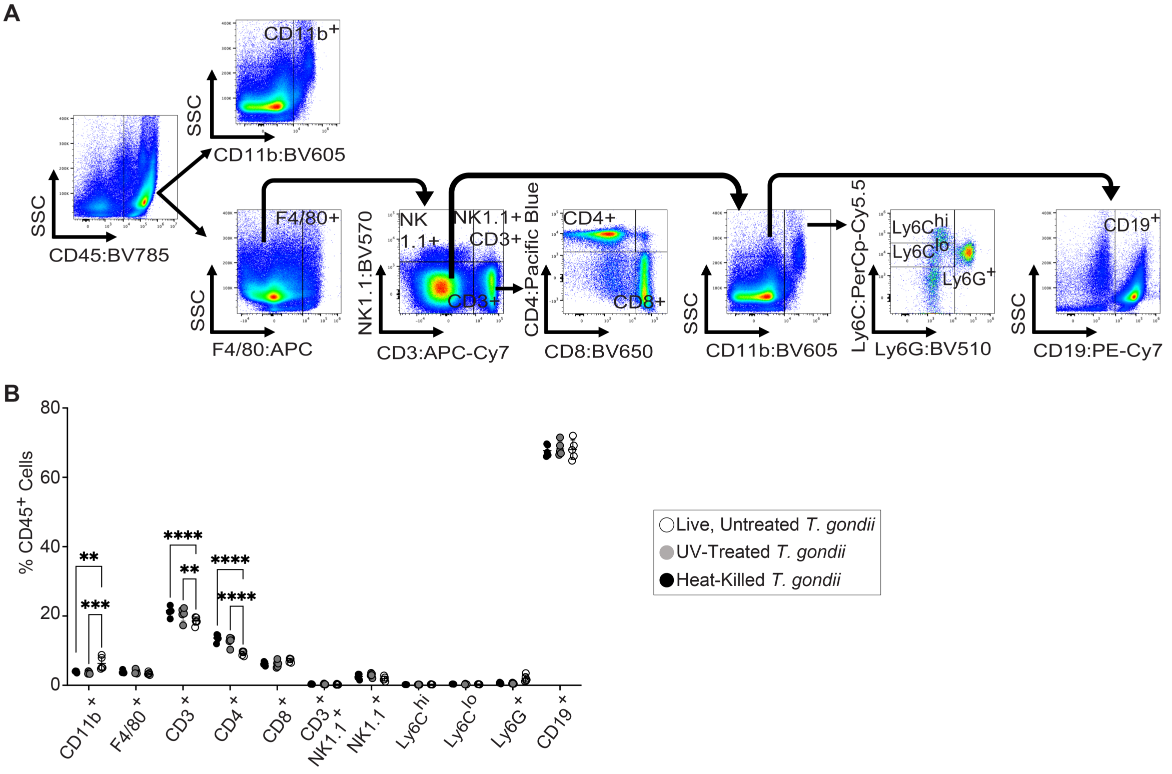

Figure S6. Impact of intracranial injection of *T. gondii* on peripheral immune responses. C57BL/6 WT mice were intracranially injected with 200 heat-killed, UV-treated, or live untreated *T. gondii*, and spleens were collected for flow cytometric analyses 7 days later. **(A)** Representative flow cytometry gating scheme of splenic immune cells from a WT mouse i.c. injected with 200 live untreated *T. gondii*. (**B**) Splenic frequencies of CD45$^+$ cells at 7 DPI. In (**B**) n = 5 mice per group. Statistical significance was determined by two-way ANOVA. **p<0.01, ***p<0.001, ****p<0.0001.

Supplementary Methods

THP-1 Cell Culture and Infection

The human monocytic THP-1 cell line was cultured in R-10% (HyClone, Logan, UT) supplemented with 2 mM L-glutamine, 100 U/ml penicillin, and 100 μg/ml streptomycin. Control or Δ*gra15* tachyzoites grown in human foreskin fibroblasts (HFF) were washed with D-10% medium and syringe lysed. Lysed parasites were washed with D-10% then passed through a 5-μm filter (EMD Millipore, Billerica, MA), and washed with D-10% medium again.
Free *T. gondii* tachyzoites were added to host cells at a multiplicity of infection (MOI) of 2. At 18 HPI, supernatants were harvested from the culture. Human IL-1β protein in the supernatant was measured using ELISA MAX Deluxe kits (BioLegend). ELISA plates were read using a Spectra Max Plus 384 plate reader (molecular Devices, San Jose, CA) using SoftMax Pro Version 5 software (molecular Devices).
